# Performance and Meat Quality of Intrauterine Growth Restricted Pigs

**DOI:** 10.3390/ani11020254

**Published:** 2021-01-20

**Authors:** Piotr Matyba, Tomasz Florowski, Krzysztof Dasiewicz, Karolina Ferenc, Jarosław Olszewski, Michał Trela, Gilbert Galemba, Mirosław Słowiński, Maria Sady, Dominika Domańska, Zdzisław Gajewski, Romuald Zabielski

**Affiliations:** 1 Center for Biomedicine Research, Center for Regenerative Medicine, Department of Large Animal Diseases and Clinic, Institute of Veterinary Medicine, Warsaw University of Life Sciences, Veterinary Research Centre, 02-797 Warsaw, Poland; pmatyba@vp.pl (P.M.); karolina_ferenc@o2.pl (K.F.); jarek.olszewski01@gmail.com (J.O.); trela_michal@wp.pl (M.T.); m.sady92@gmail.com (M.S.); domanska.dominika@gmail.com (D.D.); zgajewski@supermedia.pl (Z.G.); 2 Department of Food Technology and Assessment, Institute of Food Sciences, Warsaw University of Life Sciences, 02-776 Warsaw, Poland; tomasz_florowski@sggw.edu.pl (T.F.); krzysztof_dasiewicz@sggw.edu.pl (K.D.); miroslaw_slowinski@sggw.edu.pl (M.S.); 3 Pig Farm, 62-600 Kiełczew Smużny IV, Poland; lgalemba@wp.pl

**Keywords:** IUGR, pigs, growth performance, meat quality, sensory evaluation

## Abstract

**Simple Summary:**

Pigs with intrauterine growth restriction (IUGR) are neonates born at term but having low birth weight and a characteristic head shape. IUGR is observed in 6-10% of pig neonates. IUGR causes problems in livestock farms due to high mortality of the piglets in the first days of life and slower postnatal growth. Tracing the surviving IUGR piglets is difficult, so the data on their post-weaning growth, performance, and carcass quality is scanty. This study shows that the post-weaning performance of IUGR pigs is poorer than that of their normal littermates. However, the growers’/fatteners’ morbidity and meat quality is not different, and the consumer preference tests clearly show that the meat of the IUGR pigs is more readily accepted than that of the normal pigs. Consumers indicated better taste, smell, and consistency of this meat. The basis of consumers preference is in a slightly different chemical composition and structure of the muscle tissue. This study shows that efforts toward reducing high mortality among IUGR neonates may be beneficial.

**Abstract:**

Intrauterine growth restricted (IUGR) pigs are characterized by high perinatal mortality and dysfunction of internal organs, adipose, and muscle tissues. However, little is known about the post-weaning performance and meat quality of the IUGR pigs. The aim of this study was to compare normal pigs and pigs with IUGR from birth until slaughter, also with respect to their meat quality. Pigs with the IUGR achieved lower slaughter weight but did not differ significantly from normal pigs in terms of their meat content. The IUGR did not negatively affect the culinary quality of the obtained meat, including its content of basic chemical components and energy value, as well as hardness, chewiness, cohesiveness, elasticity, and penetration force. The meat of the IUGR pigs, when compared to the meat of normal pigs, was characterized by higher pH, lower EC (Electrical Conductivity) and drip loss; it was also tenderer and obtained higher scores in sensory evaluation of taste, smell, and general desirability. Therefore, such raw material can be appreciated by the consumers and can be used for the production of culinary portions similarly to the raw material obtained from normal pigs.

## 1. Introduction

Pig neonates displaying the intrauterine growth restriction (IUGR) are born on time but are characterized by low body weight and a characteristic shape of the facial part of their head [1,2,3]. The IUGR-associated disruption of the foetal program spares the development of vital organs, such as brain and heart, but other body organs display various degrees of modification of their structure and function due to inadequate delivery of nutrients through the placenta [4]. This phenomenon is known in the literature as the “thrifty phenotype”, first described in humans by Hales and Barker [5]. Such structure and function modifications were recently documented in neonatal pigs in the small intestine, liver, pancreas, kidneys, as well as in the skeletal muscles and adipose tissue [6,7,8,9], and resulted in life-long modification of energy metabolism. This in turn increases the animal’s susceptibility to develop metabolic syndrome [5] and makes the adult pigs with IUGR history obviously unsuitable for reproduction. However, since about 7% of all newborn piglets show the symptoms of the IUGR [4], the practical question is what to do with these piglets. IUGR pig neonates manifest poor growth, are less mobile and more prone to be crushed by the sow, and get sick much more often [3]. However, Baek and co-workers observed that moderate foetal and postnatal growth restriction has limited effect on the growth of organs, blood biochemistry, and immune cell development during the first 3 weeks after birth in preterm pigs [10]. In their study, factors leading to moderate growth restriction did not prevent the surviving preterm pigs from following a near-normal trajectory of immune development, suggesting that neonates have a remarkable capacity to adapt their systemic immune system during the first weeks after birth.

The mortality rate among IUGR piglets, especially during the neonatal and weaning periods, is significantly higher than among piglets free of the IUGR. Depending on the severity of the IUGR, a considerable number of them will not survive the first three days of life, but due to improved farm management and quality of animal handling, the number of saved low birthweight piglets, including those with IUGR, is increasing [4]. If the survival rate of the IUGR piglets was manageable, would it be economically reasonable for the farmer to grow them? And what about the quality of the meat from the IUGR pigs offered to the consumer? The last question in particular remains open, since the results obtained so far have been conflicting [11,12,13]. The aim of the present study was to evaluate the performance of IUGR vs normal pigs at slaughter, and to further evaluate their carcasses and their meat quality.

## 2. Materials and Methods

### 2.1. Animal Health Status and Nutrition

The protocol was conducted in compliance with the European Union’s regulations concerning the protection of experimental animals. Since the study protocol did not introduce any additional actions than routine farm practices, no approval by the Local Ethical Committee was necessary. The study was performed in a private commercial pig farm located in central Poland with 600 sows (pig hybrid PIC^®^: Camborough plus ♀ and PIC410 ♂) producing annually about 18,100 weaners and about 12,300 fattening pigs. The farm sells approximately 5800 growing pigs to nearby fattening farms annually. In 2019, the numbers describing the farm’s performance were as follows: Number of pigs born alive—13.92, preweaning mortality—9.4% (weaning at about day 28 postnatal), number of piglets weaned by sow—12.69, number of piglets produced per sow per year—33.13, nursery mortality—1.38%, and fattening mortality—1.9%. Regarding the health status, the herd was negative for porcine reproductive and respiratory syndrome virus and swine influenza, negative for *Actinobacillus pleuropneumoniae* (APP) as confirmed by ELISA tests in sera, and leptospirosis-negative as confirmed by a microagglutination test. The following diseases were present at the farm but controlled by vaccination/medication protocols: Circovirosis (PCV2), *Mycoplasma hyopneumoniae*, atrophic rhinitis, streptococcosis, oedema disease, enterotoxinogenic *Escherichia coli*, intestinal adenomatosis, and coccidiosis.

Pregnant sows were fed a standard wet diet (Table 1, Tables S1 and S2) on concentrate base by De Heus sp. z o.o. (Poland), and 3 weeks before farrowing the diet was switched to the wet diet for lactating sows. On postnatal day (PD) 3, all newborn piglets were injected intra-muscularly with 200 mg iron chelate (Gleptosil, Ceva). Males were castrated in the first postnatal week. From PD 7, piglets were fed creep feed ad libitum with dry pre-starter diet. Piglets were weaned on PD 28 on a commercial pre-starter wet diet (Table 1) and continued on this diet for 4–5 weeks. Pigs were then fed standard wet starter, grower and finisher diets until slaughter (Table 1). The feed and water were provided ad libitum.

### 2.2. Study Protocol

The performance of the total of 52 sows that delivered piglets between 11 and 21 February 2020 as well as their offspring was recorded. The pigs were weaned on PD 35, moved to growing/fattening sector on PD 89, and slaughtered on PD 188 at a local pig slaughterhouse. From 12 sows that farrowed in the same day (13 February 2020) pairs of littermates (one normal and one intrauterine growth restricted—IUGR) were taken for further observation until slaughter and collection of *longissimus dorsi* muscle for further analysis. IUGR piglets were recognized by low birth body weight and at least one of three characteristics listed by Hales and co-workers [3]: (1) Steep, dolphin-like forehead, (2) bulging eyes, and (3) wrinkles perpendicular to the mouth. Piglets were considered as “normal” when none of the head shape characteristics was present. All piglets in the 12 pairs (normal and IUGR) were delivered by multiparous sows at term and were clinically healthy. These pairs of piglets were marked with ear tags that were distinct from the standard orange ear tags (normal—blue, IUGR—red) for easy identification and kept with their littermates. One of the marked with red ear tag IUGR piglets died before weaning.

### 2.3. Slaughter and Meat Sampling

A total of 23 fattening pigs, 12 normal (i.e., with blue ear tags) and 11 with history of IUGR (i.e., with red ear tags), were slaughtered on PD 188 in an industrial slaughterhouse in Wielkopolska (Poland). The slaughter weight was recorded, and the carcass’ weight and meatiness were measured and classified using the SEUROP scale for grading pig carcasses just after slaughter [14]. After 24 h of incubation at +4 °C after slaughter, the carcasses of normal and IUGR pigs were dissected, and a 0.8 kg sample of the left longissimus dorsi muscle was collected from the lumbar region for further analyses. From each meat sample, a slice (weighing about 200 g) was packed into a separate foil bag to assess the size of the free leakage (see Section 2.8). All samples were transported at the temperature of the cold storage (+4 °C) to the laboratory for analysis of selected physical, chemical, and sensory properties of the tested meat.

### 2.4. Determination of Basic Chemical Composition and Energy Value

Determination of basic chemical composition (fat, protein, moisture, and collagen) was made using the method of near infrared reflectance (NIR) spectroscopy (FoodScanTM2 Meat Analyzer, FOSS Analytical, Hilleroed, Denmark) according to PN-A-82109 (2010) method. Meat samples (*m. longissimus*) weighing approximately 250 g were placed in a round measuring container made of glass and inserted into the apparatus. The principle of the operation of the apparatus consists of 16-times measurement of fat, protein, moisture, ash, connective tissue, and collagen content with the use of a monochromator with movable diffraction grating, which analyses near infrared spectrum in the 850–1050 mm range.

### 2.5. pH

The pH values were measured by the pH-meter Testo 206 pH-2 (Testo SE & Co KGaA, Titisee-Neustadt, Germany) using an electrode and a temperature sensor placed directly into the analysed raw meat (*m. longissimus*). The device was calibrated with two buffers (pH 4.0 and 7.0). All measurements were analysed in triplicates from which an average was calculated. Based on the pH measurements, the meat was classified into the RFN (red, firm and non-exudative, pH > 5.5) or exudative (PSE—pale, soft, and exudative, RSE—reddish-pink, soft, and exudative, or AM—acid meat) (pH < 5.5) categories.

### 2.6. Electrical Conductivity (EC)

The electrical conductivity (EC) of the meat was measured by Meat Quality Tester type MT-03 (Department of Microprocessor Technology EXE, Poznań, Poland). The measurement was performed by thrusting an electrode into the meat (m. longissimus) across the muscle fibres. Based on the EC measurements, the meat was classified into the exudative or the RFN category. In the classification, the accepted determining value of the criterion was established at the level of 10 mS (>10mS = exudative meat; <10 mS = RFN meat), as recommended by the producer of the device.

### 2.7. Colour Parameters and Total Colour Differences

The CIEL*a*b* (CIE—Commission Internationale de l’Eclairage, L*—lightness, a*—red/green coordinate, b*—yellow/blue coordinate) colour space parameters were determined using a Konica Minolta Chroma Meter CR-400 (Minolta, Osaka, Japan light source D65, with a measuring head hole of 8 mm). The measurements were made at a freshly sliced surface after 20 min of “blooming time”. All measurements were analysed in six replicates from which an average was calculated. Using the three-coordinate CIEL*a*b* scale, the total colour difference (ΔE) between the meats of the normal and the IUGR pigs was calculated:

ΔE = [(L*0 − L*1)2 + (a*0 − a*1)2 + (b*0 − b*1)2]0.5(1)

where: ΔE—total colour difference, L*0, a*0, b*0—means of colour parameters determined for the meat of the control pigs, L*1, a*1, b*1—means of colour parameters determined for the meat of the IUGR pigs.

In the interpretation of the results, it was assumed that: When 0 < ∆E < 1—the observer does not notice the difference; when 1 < ∆E < 2—only an experienced observer may notice the difference; when 2 < ∆E < 3.5—an unexperienced observer also notices the difference; when 3.5 < ∆E < 5—a clear difference in colour is noticed; and when 5 < ∆E—an observer notices two different colours [15].

### 2.8. Drip Loss

The drip loss was determined using meat slices weighing approx. 200 g, obtained by longitudinal dissection of muscle fibres (*m. longissimus*). Each slice was weighed and sealed in a barrier bag. The sample was stored at 4–6 °C for 24 h, the drip was decanted, and the meat was reweighed. The amount of exudation was expressed as a percent of the original weight.

### 2.9. Changes in Meat Weight during Brine Cure

Samples of meat (*m. longissimus*) weighing about 500–600 g were placed in containers with a brine solution (2:1 ratio of brine to meat). The salt (NaCl) content in the brine was selected to ensure a 1% salt concentration in the product and the resulting meat in brine sample was placed in a cold storage (4–6 °C) for 24 h. The change in the mass of the samples during brining was determined on the basis of the difference in the mass of the sample before and after brining (the result was expressed as a percentage of the initial mass of the meat).

### 2.10. Cooking Loss

Samples of meat after finishing the test with brine solution (Section 2.9) were roasted in a combi-steamer Self Cooking Centere 5 Senses (RATIONAL Aktiengesellschaft, Landsberg am Lech, Germany) at 150 °C, 60% humidity, until the meat has reached an internal temperature of 80 °C. After roasting, the samples were cooled at a temperature of +4 °C for 24 h. The cooking loss was expressed as percentage of the initial sample weight.

### 2.11. Water Holding Capacity (WHC)

Water holding capacity was determined according to the method developed by Grau and Hamm [16] using the Pohja and Niinivaara [17] modification.

### 2.12. Textural Parameters of Meat after Cooking

The textural parameters of the samples were measured using a texturometer Zwick 1120 (Zwick GmbH & Co., Ulm, Germany) at 20 °C. The cutting and penetration force were measured, and a texture profile analysis (TPA) was performed. The material for texture analysis consisted of meat samples (m. longissimus) after heat treatment described in Section 2.10. Before the measurement, the samples were conditioned at 20 °C to equalize the temperature. The samples for the cutting force measurements were cubes with dimensions of 10 × 10 × 50 mm. The cut was made across the muscle fibres using a Warner–Bratzler attachment with a flat knife. The measured parameter was the maximum force needed to cut the sample with a speed of 50 mm/min. Twenty millimeter-thick slices were used for the measurements of the penetration force. These measurements were done using a metallic, flat-felled mandrel (ø 13 mm), immersing to a depth of 10 mm with a speed of 50 mm/min. The test for texture profile analysis (TPA) was performed on the cubic meat samples (side 2 cm), which were deformed (30%) with 50 mm/min speed applied perpendicularly to the direction of the muscle fibres. The cohesiveness, springiness, hardness, and chewiness of the meat samples were calculated according to manufacturer’s instruction. Three measurements were made for each meat sample, taking the average value as the result.

### 2.13. Sensory Analysis

Sensory analysis of the meat portions was carried out using the point method. The test samples were about 3 mm thick slices of roasted meat (m. longissimus, roasting at 150 °C, 60% humidity, to an internal temperature of 80 °C). Before the analysis, the samples were conditioned at 20 °C for 30 min. A group of trained panellists conducted the scoring. Different levels of quality or intensity of sensory descriptors (colour, taste, smell, consistency, and overall assessment) of the evaluated meat samples were assigned specific numerical values from 1 to 5, where 1 was the lowest and 5 was the highest score [18].

### 2.14. Histology Analysis

The microstructure of the meat was determined using light microscopy. The *longissimus dorsi* samples were fixed in 4% buffered formaldehyde and then stored in ethanol. The samples were embedded in paraffin, sliced into 5 µm sections and mounted on glass microscope slides. Deparaffinization of the slides encompassed two washes for 15 min in xylene and rehydration in decreasing concentrations of ethanol (from 100% to 70%). Serial histological 5 µm sections were stained with haematoxylin and eosin for morphometric analysis under the light microscope. The morphometric analysis included histometry of the muscle fibres and the adipose cells. Five to ten slides were prepared for each tissue sample and 30 specimen measurements were performed using an optical binocular microscope (Olympus BX60; Olympus, Warszawa, Poland) coupled via a digital camera to a personal computer equipped with a cellSens Standard 1.9 (Olympus) software.

The quantitative analysis of the histological structure of the examined muscles was performed using the TissueFAXS PLUS system (TissueGnostics, Vienna, Austria). Large-area histological slides (~500,000 μm^2^) stained by Masson’s trichrome were archived entirely as high-resolution photos using a dry ×20 lens. On their basis, a tissue scanner automatically reproduced a microscopic photograph covering the entire analysed histological section. The HistoQuest image analysis software (TissueGnostics, Austria) characterized 3 colour palettes corresponding to muscles, connective tissue fibres, and free spaces in the specimen. Using the algorithms of the image analysis software, the photo surface was divided into regions with the same colour palette. The regions were characterized in terms of a number of geometrical features such as area, maximum width, maximum thickness, and perimeter. The output data was the sum of the area of each of the 3 markers in the preparation, the average circumference and area of the individual fibres, and the average maximum width and thickness of the individual muscle fibres. A prefilter was set in the analysis to exclude any non-specifically detected objects. This filter rejected from the analysis fibres smaller than 200 and larger than 5000 square micrometres.

### 2.15. Statistical Analysis

The results were subjected to a two-stage statistical analysis. In the first step, we checked the uniformity of the SD and the Kolmogorov–Smirnov-A test of the normal distribution. Depending on the outcome, the test data were then analysed using an unpaired t-test or either the t-test and Welch (normal distribution) or Mann–Whitney test-A (in its absence) tests. All statistical analyses were done using the GraphPad Prism v.5.0 (GraphPad Software, San Diego, CA, USA); *p* < 0.05 was considered as significant, *p* < 0.01 as highly significant, and *p* < 0.1 as a trend.

## 3. Results

### 3.1. Pig Performance

The examined sows (*n* = 52) delivered a total of 779 liveborn piglets, including 720 normal neonates (mean birthweight ± standard deviation: 1.42 ± 0.28 kg, min-max: 0.6–2.5 kg), 59 with IUGR (mean birthweight ± standard deviation: 0.57 ± 0.11 kg, min-max: 0.4–0.8 kg), and 63 deadborn piglets (55 without and 8 with IUGR). The proportion of the IUGR piglets that were born alive to all born alive piglets was 7.57%. In the group of 52 sows, only 4 were primiparous, while the other were multiparous (i.e., 2–10 pregnancies). The primiparous sows delivered no IUGR (dead or alive) piglets, while each multiparous sow delivered, on average, 1.25 piglets with the IUGR. The mortality rate of the pigs before weaning was 11.0% and from weaning to slaughter was 1.71%. The majority of pigs that died before weaning displayed the IUGR. In contrast, after weaning there were no differences in the proportion of deaths between normal and IUGR pigs. Table 2 shows the mean body weights at birth, weaning (PD 35), transfer to fattening sector (PD 89), and slaughter (PD 188), as well as wet carcass weight and meatiness among the 12 normal and 11 IUGR pigs. The body weight between pairs was significantly different at birth, after which the IUGR piglets caught-up with their body weight, but a significant body weight difference was again observed at slaughter. After weaning, the pigs were kept in groups, each consisting of 4 to 5 litters; therefore, it was not possible to measure individual feed consumption nor to compare the individual feed consumption of the control pigs and the pigs with the IUGR. During the growing–finishing period the calculated feed conversion ratio (FCR) was 2.45 and the daily body weight gain was about 910 g.

There was a 15 kg difference between the normal pigs and the pigs born with the IUGR in the wet weight of the carcasses, but no statistically significant differences were seen in their meatiness (Table 2). The population of pigs with the IUGR was characterized by a slightly higher percentage of pigs placed in the U meatiness class (27 and 17%, respectively) when compared to pigs from the control group. According to the SEUROP meatiness scale, in the normal group 3 pig carcasses were in class S (the highest class of meat), 7 in class E, and 2 in class U (the lowest class of meat), while among the IUGR pigs 3 carcasses were in class S, 5 in class E, and 3 in class U. In addition, the muscle *longissimus dorsi* tended to have a smaller surface area in pigs with the IUGR as compared to normal pigs (Table 2). It is noteworthy, however, that we have found very large variation in the area of m. longissimus in both populations, i.e., from 42.7 to 78.4 cm^2^ in the normal group and from 32.1 to 73.0 cm^2^ in the IUGR group.

### 3.2. Chemical Composition and Energy Value of Meat

There was no statistically significant effect of the IUGR on the basic chemical composition of the meat (*m. longissimus*), including water, protein, fat, and ash content. The meat obtained from the pig carcasses of both groups did not differ in energy value (Table 3), but statistically significant differences were found in the collagen content, and consequently also in the connective tissue. The longissimus muscles from the pigs with the IUGR were characterized by a significantly higher collagen and connective tissue content, which may be a differentiating factor with respect to their technological and culinary quality.

### 3.3. Colour Parameters of Meat

It was found that the meat (*m. longissimus*) obtained from IUGR pigs was characterized by a significantly lower values of the b* colour parameter than that of normal pigs (Table 4). Moreover, there was a tendency towards slightly lower values of the L* colour parameter and lower values of the a* colour parameter of such meat. The meat of the IUGR pigs was therefore slightly darker, less red and less yellow in colour than that of the normal pigs. Based on the calculated parameter of the total colour difference, it was found that the difference in the colour of meat obtained from the normal and IUGR pigs was visible to the unexperienced observer.

### 3.4. pH, Eectrical Conductivity, and Drip Loss of Meat during Storage and Roasting

The mean pH values of the meat (*m. longissimus*) of both groups of pigs were typical of a good quality RFN meat. However, the meat obtained from pigs with the IUGR was found to be characterized by a significantly higher pH (by an average of 0.11 pH units) than meat from the normal pigs (Table 4). In addition, it has been found that 16.6% of the normal pigs’ meat samples had a pH < 5.5, which may indicate a PSE, RSE, or AM defect. Such meat was not found in the samples obtained from the IUGR pigs. The significantly lower electrical conductivity (EC_24h_) values also prove that the meat obtained from IUGR pigs has higher quality than the meat of the normal pigs. It was found that in this group of meat, 18.2% of the samples had electrical conductivity higher than 10 mS/cm, while in the control group it was 66.6% of the samples. Both the higher pH_24h_, and the lower rate of post-slaughter glycolysis in the first hours after slaughter (as is indicated by lower electrical conductivity values), observed in the case of the meat of the IUGR pigs, correlated with better water holding during refrigerated storage (lower drip loss) and a tendency to greater weight gain during brining. The IUGR meat had, however, a tendency to slightly greater weight loss during heat treatment. This effect may be the consequence of the lower weight loss from the IUGR meat during the refrigerated storage, resulting in a higher initial water content in the meat portions before the roasting.

### 3.5. Textural Parameters of Meat after Cooking

The analysis of texture revealed a tendency of the meat (*m. longissimus*) of IUGR pigs to be slightly more tender than the meat of the normal pigs. This is indicated by the slightly lower shear force values determined during the test involving cutting across the muscle fibres of the samples (Table 5). However, no significant differences between the IUGR and the normal pigs were found in the remaining analysed meat texture parameters, i.e., those parameters that were measured when the force applied during the test acted along the muscle fibres, causing them to separate.

### 3.6. Sensory Quality

The sensory quality analysis conducted on the meat from pigs of both groups after the heat treatment showed higher culinary quality of the meat from the IUGR pigs (Table 5). Significant differences were found in the evaluation of smell, taste, and texture, as well as in the overall evaluation. The panellists found no statistically significant differences only in the case of colour, which in this case of heat-treated meat was most likely due to the degradation of the haem dyes. The higher scores obtained for the overall quality of the meat from the IUGR pigs than for the meat from normal pigs could relate to, among other things, the differences in the meats’ tenderness. As shown by the instrumental analyses, the meat of the IUGR pigs was slightly more tender, which is probably reflected by the higher scores in the consistency assessment and, consequently, in the overall quality assessment.

### 3.7. Longissimus dorsi Ultrastructure

**Figure 1:**
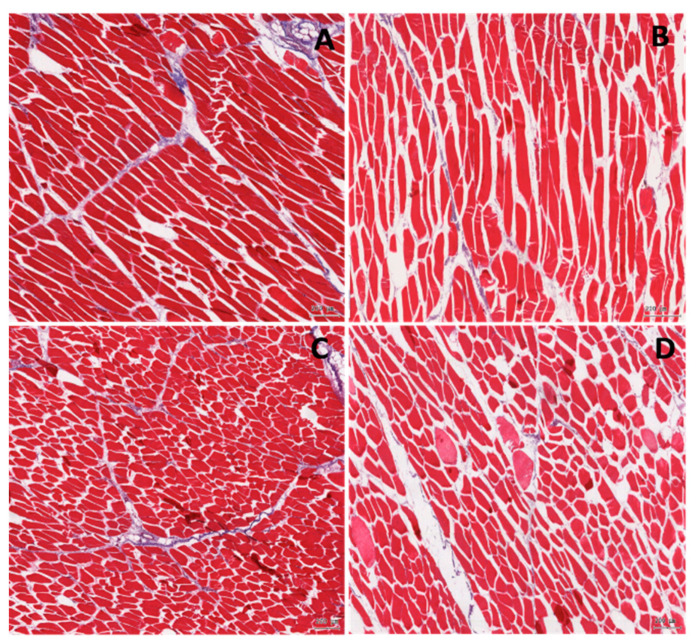
Histological analysis of the muscle in the normal (**A,C**) and the intrauterine growth restricted, IUGR (**B,D**) pigs. Longitudinal (**A,B**) and transverse (**C,D**) section. In the IUGR muscle, the empty spaces between the muscle cells are significantly larger. Distinctly more gain muscles are observed in the IUGR samples.

Histology analyses of the meat samples showed normal structure of the longissimus dorsi muscle in both examined groups (Table 6). However, the number of giant fibres was several-fold higher in the IUGR pigs as compared to the normal pigs. The giant fibres had a relatively large cross section area of the myocytes and a lightly pink stained cytoplasm (Figure 1).

There were no significant differences in the proportion of the myocytes, the connective, and the adipose tissues between the two groups, but in the samples from the IUGR pigs, there was a tendency toward a lower proportion of the myocytes. The other cell components of the *longissimus dorsi* muscle (e.g., the blood and lymph capillary vessels and the nerve fibres) contributed to no more than 2% of the field of vision. This was similar in the normal and in the IUGR pigs, therefore the split between the cells was remarkably larger in the slides from the IUGR’s samples when compared to the normal pigs (Table 6). The *longissimus dorsi* myocytes of the IUGR pigs showed shorter myocyte perimeter and length as compared to the normal pigs (Table 6).

## 4. Discussion

In the examined farm, 7.5% of all live-born pig neonates showed signs of the IUGR. The mortality of the pigs with the IUGR was the highest in their first 2 weeks of life (mainly in the first 2–3 postnatal days), and starting from 2 weeks after weaning it did not differ from normal pigs. In the present study, we were not able to confirm if the growing/fattening pigs with the IUGR were more susceptible to diseases; whether they could potentially be at increased risk of transmitting diseases, and/or constitute a disease reservoir. However, Amdi et al. [19] observed a moderate suppression of the immune response of IUGR piglets on PD 24 which may have implications for resistance to pathogen challenges in the post-weaning period. Pig neonates with the IUGR were on average significantly lighter at birth than the normal pigs, though there were about 12% of neonates with birth body weight below 1.0 kg (small for gestational age (SGA)) that did not show the signs of the IUGR according to the head anatomical criteria given elsewhere [1,2,3]. However, the postnatal mortality of the SGA pigs was not as high as that of the IUGR pigs, and it was within the range of all the non-IUGR pigs. Our results are in contrast to the results of Wu and co-workers [20], who suggested a much higher frequency of IUGR pigs (15–20%) in a pig population. Using the birth body weight and the head anatomy criteria [1,2,3], we may presume to be better able to distinguish the IUGR piglets from the SGA piglets that do not manifest the signs of the IUGR. Nevertheless, during the first half of the production cycle (growing), there were no weight differences between the non-IUGR and the IUGR pigs which is in contrast with results reported by Lynegaard and co-workers [21]. In their study, IUGR pigs showed smaller average daily gains and required 6 additional days to reach a body weight of 30 kg in comparison to normal pigs. The discrepancy may be related to a smaller size of experimental groups and high animal to animal variation in the body weight in our IUGR pigs. However, in our study the differences in the body weight reappeared in the finishing period resulting in a net difference of approximately 15 kg in the slaughter body weight, indicating that the IUGR depresses protein deposition and body growth in the finishing pigs and/or lower feed intake (but a similar FCR). Poor protein deposition will result in increased FCR and higher feed costs, whereas lower feed intake will take longer to reach the desired slaughter weight thereby disturbing pig flow and herd management. More studies are needed to better understand the growth performance characteristics of IUGR growing and fattening pigs. Therefore, it is of no great benefit to the farmer to grow the IUGR pigs that display poor meat and fat deposition in the finishing period. On the other hand, the overall average daily feed intake (ADFI) = 2.2 kg, and feed conversion ratio (FCR) = 2.45 (all pigs collectively, including IUGR) during the growing–finishing period warrants a profit to the farmer due to a good overall performance of the pigs (slaughter weight, meatiness, carcass quality). On the other hand, the results of the meat evaluations were unexpected and showed that the characteristics of the meat from the IUGR pigs showed a number of parameters that makes it alike, or even better, for cooking than the meat of the normal pigs, which was further confirmed by the consumer tests.

The pigs with the IUGR had a lower slaughter weight and subsequently a lower weight of the carcass as a response to the thrifty phenotype during the foetal period [5]. Nevertheless, the IUGR pigs which survived the early postnatal period and weaning did not show in the further phases, growing and fattening, a tendency toward higher susceptibility to disease as compared to normal pigs, and the obtained IUGR pig carcasses were characterized by a meatiness similar to that of normal pigs. This is an important information for farmers and the meat industry. The currently practiced system of settlements between the farmers and the meat processing plants, which takes into account the meatiness of pigs, makes farmers interested in producing livestock with the highest meatiness. The high meat content of the pigs is also a desirable feature for the industry, resulting in a leaner meat for the culinary and processing purposes. A history of IUGR did not negatively affect the culinary quality of the obtained meat. Like the meat of the normal pigs, the portions of pork loin obtained from the IUGR pigs were characterized by a low-fat content, which is desired by the modern consumers [22,23,24,25]. The slightly higher collagen content found in the meat of the IUGR pigs could be the result of a smaller circumference of the muscle fibres. However, the differences were small, and this feature did not have a negative impact on other characteristics of the culinary quality of the meat.

Apart from the tissue composition, the amount of drip loss is an important distinguishing quality feature of culinary meat. Free leakage accumulating in the packaging of commercial portions of meat significantly reduces their consumer desirability [23,24] and constitutes a good medium for the development of microorganisms [26]. The lower amount of free leakage found in the meat obtained from the IUGR pigs is in this context an extremely desirable feature. The lower free leakage from the meat of the IUGR pigs could be related to the slower post-slaughter glycolysis rate, as evidenced by the lower electrical conductivity values. This feature indirectly indicates the adverse changes occurring in the tissues during a too fast post-slaughter glycolysis, which later translates into the occurrence of a PSE-like quality defect [27,28]. Another factor that could have resulted in the lower drip loss and greater weight gain during brining of the IUGR meat, as compared to that of the normal pigs, was the higher pH of the meat (i.e., further from the isoelectric point of the muscle proteins). As a result, the meat was characterized by a greater water-holding capacity.

Another feature that may have been influenced by the slower course of the post-slaughter glycolysis and the higher pH of the meat of the IUGR pigs was the colour of the meat. The faster the rate of the post-slaughter glycolysis in the first hour after slaughter and the lower the final pH of the meat, the brighter was its colour. Such effect is found, for example, in the PSE meat [29,30]. The obtained results of the pH, electrical conductivity, drip loss, and meat colour analyses may also indicate that the pigs burdened with the IUGR may have lower susceptibility to ante mortem stress, and thus a lower risk of such occurrences as a PSE-like meat. Many authors [28,31,32,33,34] indicate the importance of stress in the formation of a PSE defect.

The lack of a significant impact of IUGR on most of the analysed texture parameters, i.e., the penetration force, cohesiveness, springiness, hardness, and chewiness resulted from the lack of visible disorders in the muscle structure, although muscle pathologies, such as numerous giant myocytes, were present in the meat of the IUGR pigs. The meat of the IUGR and normal pigs was also characterized by a similar content of intramuscular fat, i.e., the ingredient that has a particularly significant effect on the texture of the meat [35,36]. At the same time, it was found that the meat obtained from the IUGR pigs exceeded the quality of the meat of normal pigs in terms of tenderness. The basis for these differences can be found in the slower rate of post-slaughter changes and, presumably, in the shorter muscle fibres. This greater tenderness of meat from the IUGR pigs translated into a higher sensory quality, i.e., higher scores awarded for, among others, consistency, and therefore for overall quality. Concluding, the obtained results indicate that the meat of the pigs with the IUGR can be used to prepare portions of dinner dishes with a quality comparable to, or even slightly better than, that of the meat of normal animals.

## 5. Conclusions

Following strong selection in the early postnatal period, the surviving IUGR pigs showed number of risks including high early postnatal mortality, and reduced performance in the nursing and fattening phase. However, the susceptibility to diseases of growing and fattening IUGR pigs did not differ from that in normal pigs. Despite the lower carcass weight, the quality of meat of the IUGR pigs showed a number of positive traits, including consumer preference tests, which were supported by some chemical, physical, and morphology analyses.

## Figures and Tables

**Table 1:** Feeding protocol of pregnant and lactating sows and their offspring.

Group	Water/Whey/Dry Feed (%)	Dry Matter (%)	Metabolizable Energy (MJ/kg)	Crude Protein (%)
Pregnant sows	39.5/42.0/18.5	88.00	9.50	12.83
Lactating sows	36.8/40.0/23.2	88.00	9.84	16.04
Creep feed	Dry feed	92.76	10.99	19.43
Pre-starter (<30 kg BW)	72.4/27.6 ^a^	88.00	12.81	17.01
Starter (30–60 kg BW)	36.0/39.0/25.0	88.00	13.52	16.42
Grower (60–90 kg BW)	36.4/37.0/26.6	88.00	13.17	16.37
Finisher (>90 kg BW)	31.0/41.0/28.0	88.00	12.78	15.65

^a^ water and dry feed only (no whey); BW—body weight.

**Table 2:** Body and carcass weights, meatiness, and longissimus dorsi muscle surface area of the normal (*n* = 12) and intrauterine growth restricted (*n* = 11) pig pairs from birth to slaughter. Mean ± SD, Student *t*-test.

Item	Normal	Intrauterine Growth Restricted	*p*-Value
Birth weight (kg)	1.35 ± 0.19	0.65 ± 0.07	0.001
Weaning weight (kg)	5.13 ± 0.39	5.00 ± 0.86	0.651
Transfer to fattening weight (kg)	31.78 ± 4.39	27.96 ± 7.30	0.142
Slaughter weight (kg)	118.56 ± 9.0	99.32 ± 9.76	0.001
Carcass wet weight (kg)	92.48 ± 7.72	77.47 ± 7.61	0.001
Meatiness (%)	58.01 ± 3.22	57.72 ± 3.66	0.841
*Longissimus dorsi* muscle surface area (cm^2^)	59.4 ± 10.3	51.0 ± 9.7	0.071

**Table 3:** Chemical composition and energy value of meat of normal (*n* = 12) and intrauterine growth restricted (*n* = 11) pigs. Mean ± SD, Student *t*-test.

Parameter	Normal	Intrauterine Growth Restricted	*p*-Value
Water (%)	73.38 ± 1.00	73.36 ± 0.74	0.851
Protein (%)	23.7 ± 0.5	23.4 ± 0.4	0.221
Collagen (%)	0.56 ± 0.06	0.64 ± 0.05	0.001
Connective tissue (%)	2.36 ± 0.27	2.75 ± 0.24	0.010
Fat (%)	2.31 ± 1.09	2.46 ± 0.80	0.723
Asch %	1.03 ± 0.03	1.04 ± 0.03	0.854
Energy value (kcal)	116 ± 9	116 ± 7	0.911

**Table 4:** Colour parameters and physical analysis of the meat of the normal (*n* = 12) and intrauterine growth restricted (IGUR) (*n* = 11) pigs. Mean ± SD, Student *t*-test.

Parameter	Normal	Intrauterine Growth Restricted	*p*-Value
Colour parameter L*	52.88 ± 2.24	50.61 ± 3.16	0.070
Colour parameter a*	7.09 ± 0.53	6.42 ± 1.14	0.094
Colour parameter b*	4.93 ± 0.98	3.68 ± 0.54	0.002
pH_24h_	5.55 ± 0.07	5.66 ± 0.08	0.002
EC_24h_ (mS/cm)	10.2 ± 0.7	9.4 ± 0.9	0.046
Drip loss (%)	4.3 ± 1.2	2.6 ± 0.8	0.001
Weight gain during brining (%)	2.2 ± 0.4	2.6 ± 0.7	0.108
Cooking loss (%)	46.0 ± 2.4	48.1 ± 3.7	0.117

* ΔE_normal-IUGR_ = 2.67; EC—electrical conductivity.

**Table 5:** Texture and sensory evaluation of meat of the normal (*n* = 12) and intrauterine growth restricted (*n* = 11) pigs. Mean ± SD, Student-*t* test.

Parameter	Normal	Intrauterine Growth Restricted	*p*-Value
Shear force (N/cm^2^)	46.0 ± 11.2	37.0 ± 8.7	0.055
Penetration force (N)	54.2 ± 9.7	57.2 ± 13.1	0.543
Cohesiveness	0.39 ± 0.05	0.39 ± 0.03	0.642
Springiness	0.61 ± 0.05	0.62 ± 0.03	0.579
Hardness (N)	51.1 ± 12.3	57.4 ± 10.5	0.300
Chewiness (N)	12.6 ± 4.0	13.9 ± 3.3	0.456
Sensory: Colour (points)	4.6 ± 0.2	4.7 ± 0.3	0.382
Taste (points)	4.1 ± 0.2	4.6 ± 0.3	0.001
Smell (points)	4.3 ± 0.2	4.7 ± 0.2	0.001
Consistency (points)	4.2 ± 0.2	4.7 ± 0.3	0.001
Overall assessment (points)	4.2 ± 0.2	4.7 ± 0.3	0.001

**Table 6:** Histometric analysis of the ultrastructure of the *longissimus dorsi* muscle and single myocyte of the normal (*n* = 12) and intrauterine growth restricted (*n* = 11) pigs. The percentage of myocytes, connective tissue, and adipocytes in the field of vision (values do not add to 100% because of the 1–2% of the blood and lymphatic vessel cells and nerve fibres and the split between the groups of muscle fibres), and ratios between the cell types. Mean ± SD, Student-*t* test.

Parameter	Normal	Intrauterine Growth Restricted	*p*-Value
Myocytes (%)	65.7 ± 1.7	56.4 ± 10.8	0.121
Connective tissue (%)	4.7 ± 1.1	3.4 ± 1.5	0.200
Adipocytes (%)	4.5 ± 0.9	4.0 ± 1.4	0.433
Split (%)	22.7 ± 2.9	34.1 ± 10.4	0.041
Connective tissue to myocytes ratio	0.072 ± 0.025	0.066 ± 0.033	0.760
Adipocytes to myocytes ratio	0.069 ± 0.013	0.075 ± 0.032	0.707
Myocyte: Perimeter (μm)	282 ± 65	206 ± 55	0.026
area (μm^2^)	2024 ± 462	1882 ± 380	0.510
length (μm)	87.9 ± 12.2	76.1 ± 7.8	0.048
width (μm)	34.8 ± 2.4	35.8 ± 2.6	0.451

## Data Availability

The data presented in this study are available on request from the corresponding author.

## Supplementary Materials

The following are available online at https://www.mdpi.com/2076-2615/11/2/254/s1, Table S1: Feed composition (%) for different production groups; Table S2: Composition (%) of the concentrate for sows and pigs.
