# Supplementary material for: Performance and Meat Quality of Intrauterine Growth Restricted Pigs
Source: Animals (Basel). 2021 Jan 20;11(2):254. doi: 10.3390/ani11020254 (PMC7909567; doi:10.3390/ani11020254)
Supplement: Supplementary file 1 [file animals-11-00254-s001.pdf]

Supplement

Table S1. Feed composition (%) for different production groups.

	Pregnant sow	Lactating sow	Pre-starter (weaning to 30 kg BW)	Starter (30-60 kg BW)	Grower (60-90 kg BW)	Finisher (>90 kg BW)
Water	39.3	38.0	71.8	36.0	34.7	31.0
Whey	42.0	37.3	0.0	39.0	42.0	41.0
Concentrate[1]	5.2	6.6	8.4	3.7	2.9	3.8
Maize grain silage	0.0	0.0	0.0	3.5	4.8	10.8
Wheat	1.2	7.5	11.5	9.0	7.8	0.0
Barley	8.5	6.8	6.6	8.8	7.8	6.7
Triticale	3.8	3.8	1.5	0.0	0.0	6.8

[1]concentrate composition is given in Table 2.
BW – body weight.

Table S2. Composition (%) of the concentrate for sows and pigs.

	Pregnant sow	Lactating sow	Pre-starter (from weaning to 30 kg BW)	Starter, Grower and Finisher[1] (>30 kg BW)
Total protein (%)	13.25	16.05	18.06	15.38
Fat (%)	3.67	3.91	4.50	2.02
Crude fiber (%)	6.41	4.50	3.75	4.38
Ash (%)	5.07	5.60	4.97	5.38
Dry matter (%)	88.00	88.00	88.00	88.00
Starch (%)	35.73	38.38	41.21	40.36
Lactose (%)	5.39	3.79	1.51	3.88
Lysine (%)	0.61	0.92	1.23	1.06
Methionine (%)	0.21	0.29	0.42	0.31
Met+Cyst (%)	0.49	0.61	0.74	0.60
Threonine (%)	0.50	0.63	0.80	0.67
Tryptophan (%)	0.17	0.20	0.24	0.19
Ca (%)	0.70	0.95	0.60	0.65
P total (%)	0.40	0.45	0.51	0.45
Na (%)	0.26	0.20	0.22	0.24
Vit A (IU/kg)	12044	12002	12542	6652
Vit D_3 (IU/kg)	2007	2000	2006	1109
Vit E (mg/kg)	150.7	200.0	100.3	100

[1]Vitamins: vit. C - 67 mg/kg, vit. K – 3.77 mg/kg, vit. B_1 – 2.22 mg/kg, vit. B_2 – 5.54 mg/kg, vit. B_6 – 3.99 mg/kg, vit. B12 – 39 µg/kg, nicotinic acid – 26.61 mg/kg, panthotic acid – 13.86 mg/kg, folic acid – 2.77 mg/kg, biotin – 133 µg/kg, choline chloride 332.62 mg/kg; minerals: Zn – 88.96 mg/kg, Fe – 79.97 mg/kg, Mn – 44.78 mg/kg, Cu – 12.66 mg/kg, J - 1.55 mg/kg, Se – 0.40 mg/kg.
BW – body weight.
